# Relationship between seasonal respiratory allergies and cashew's pollination period in students at a university of northeast Brazil

**DOI:** 10.1186/1939-4551-8-S1-A114

**Published:** 2015-04-08

**Authors:** Fabiane Pomiecinski, Ana Luiza Mapurunga Gonçalves, Karen Adria Romão De Lima, Mariana Aquino Holanda Pinto, Mariana Lustosa Farias Barros, Sarah Gomes Peixoto

**Affiliations:** 1 Universidade De Fortaleza – Unifor, Brazil

## Background

Observe if students exposed to pollen cashew realize worsening of their symptoms in the pollen season.

## Methods

Cross-sectional and descriptive study aimed at medical students of a university in the Northeast, exposed to pollen cashew. An electronic questionnaire about allergic rhinitis, allergic conjunctivitis, asthma and perception regarding seasonality and aggravating factors was made available for free participation in a site of the institution.

## Results

The questionnaire was answered by a total of 108 students, aged 16-40 years, 80% (84) between 19-25 years. Of the total, 68% (73) reported symptoms of allergic rhinitis, and 70% (52) intermittent rhinitis and 65% (48) had moderate to severe. 40% (43) reported having allergic conjunctivitis, intermittent was the majority (77%), being ocular pruritus the main symptom. Of the total, 17% (18) said they experience symptoms of asthma, 75% intermittent asthma. 33% had worsening of symptoms in the second half of the year. As to the triggering factors, about 30% reported worsening with dust and mold, and 8% believe that pollination of cashew aggravates their symptoms.

## Conclusions

The prevalence of allergic rhinitis (68%) and asthma (17%) was above that found in the general population is 30% and 12% respectively, however, we have to take into account the selection bias. Most realize worsens with mites and fungi, as in the general population. It is interesting that 35% reported worsening in the second half, of the year the pollen season, but only 8% believe that worsen due to pollination of cashew. It seems that if pollen of cashew is actually an allergen, it would not be the only one. The cashew is considered a heavy pollen which could not travel long distances, but on our campus there are a large number of cashew trees. Moreover, at this time of year, we get wind and thus more likely mites and fungi. Thus, more data are needed to establish the relation of cause and effect but, this study paves the way for others to assess if the pollen cashew is a villain or even a myth in our city.

